# Structural insights into the regulatory mechanism of the *Pseudomonas aeruginosa* YfiBNR system

**DOI:** 10.1007/s13238-016-0264-7

**Published:** 2016-04-25

**Authors:** Min Xu, Xuan Yang, Xiu-An Yang, Lei Zhou, Tie-Zheng Liu, Zusen Fan, Tao Jiang

**Affiliations:** National Laboratory of Biomacromolecules, Institute of Biophysics, Chinese Academy of Sciences, Beijing, 100101 China; Chinese Academy of Sciences Key Laboratory of Infection and Immunity, Institute of Biophysics, Chinese Academy of Sciences, Beijing, 100101 China; University of Chinese Academy of Sciences, Beijing, 100049 China

**Keywords:** the YfiBNR system, c-di-GMP, Vitamin B6, L-Trp, peptidoglycan layer, bioflim formation

## Abstract

YfiBNR is a recently identified bis-(3’-5’)-cyclic dimeric GMP (c-di-GMP) signaling system in opportunistic pathogens. It is a key regulator of biofilm formation, which is correlated with prolonged persistence of infection and antibiotic drug resistance. In response to cell stress, YfiB in the outer membrane can sequester the periplasmic protein YfiR, releasing its inhibition of YfiN on the inner membrane and thus provoking the diguanylate cyclase activity of YfiN to induce c-di-GMP production. However, the detailed regulatory mechanism remains elusive. Here, we report the crystal structures of YfiB alone and of an active mutant YfiB^L43P^ complexed with YfiR with 2:2 stoichiometry. Structural analyses revealed that in contrast to the compact conformation of the dimeric YfiB alone, YfiB^L43P^ adopts a stretched conformation allowing activated YfiB to penetrate the peptidoglycan (PG) layer and access YfiR. YfiB^L43P^ shows a more compact PG-binding pocket and much higher PG binding affinity than wild-type YfiB, suggesting a tight correlation between PG binding and YfiB activation. In addition, our crystallographic analyses revealed that YfiR binds Vitamin B6 (VB6) or L-Trp at a YfiB-binding site and that both VB6 and L-Trp are able to reduce YfiB^L43P^-induced biofilm formation. Based on the structural and biochemical data, we propose an updated regulatory model of the YfiBNR system.

## INTRODUCTION

Bis-(3’-5’)-cyclic dimeric GMP (c-di-GMP) is a ubiquitous second messenger that bacteria use to facilitate behavioral adaptations to their ever-changing environment. An increase in c-di-GMP promotes biofilm formation, and a decrease results in biofilm degradation (Boehm et al., 2010; Duerig et al., 2009; Hickman et al., 2005; Jenal, 2004; Romling et al., 2013). The c-di-GMP level is regulated by two reciprocal enzyme systems, namely, diguanylate cyclases (DGCs) that synthesize c-di-GMP and phosphodiesterases (PDEs) that hydrolyze c-di-GMP (Kulasakara et al., 2006; Ross et al., 1991; Ross et al., 1987). Many of these enzymes are multiple-domain proteins containing a variable N-terminal domain that commonly acts as a signal sensor or transduction module, followed by the relatively conserved GGDEF motif in DGCs or EAL/HD-GYP domains in PDEs (Hengge, 2009; Navarro et al., 2011; Schirmer and Jenal, 2009). Intriguingly, studies in diverse species have revealed that a single bacterium can have dozens of DGCs and PDEs (Hickman et al., 2005; Kirillina et al., 2004; Kulasakara et al., 2006; Tamayo et al., 2005). In *Pseudomonas aeruginosa* in particular, 42 genes containing putative DGCs and/or PDEs were identified (Kulasakara et al., 2006). The functional role of a number of downstream effectors of c-di-GMP has been characterized as affecting exopolysaccharide (EPS) production, transcription, motility, and surface attachment (Caly et al., 2015; Camilli and Bassler, 2006; Ha and O’Toole, 2015; Pesavento and Hengge, 2009). However, due to the intricacy of c-di-GMP signaling networks and the diversity of experimental cues, the detailed mechanisms by which these signaling pathways specifically sense and integrate different inputs remain largely elusive.

Biofilm formation protects pathogenic bacteria from antibiotic treatment, and c-di-GMP-regulated biofilm formation has been extensively studied in *P. aeruginosa* (Evans, 2015; Kirisits et al., 2005; Malone, 2015; Reinhardt et al., 2007). In the lungs of cystic fibrosis (CF) patients, adherent biofilm formation and the appearance of small colony variant (SCV) morphologies of *P. aeruginosa* correlate with prolonged persistence of infection and poor lung function (Govan and Deretic, 1996; Haussler et al., 1999; Haussler et al., 2003; Parsek and Singh, 2003; Smith et al., 2006). Recently, Malone and coworkers identified the tripartite c-di-GMP signaling module system YfiBNR (also known as AwsXRO (Beaumont et al., 2009; Giddens et al., 2007) or Tbp (Ueda and Wood, 2009)) by genetic screening for mutants that displayed SCV phenotypes in *P. aeruginosa* PAO1 (Malone et al., 2012; Malone et al., 2010). The YfiBNR system contains three protein members and modulates intracellular c-di-GMP levels in response to signals received in the periplasm (Malone et al., 2010). More recently, this system was also reported in other Gram-negative bacteria, such as *Escherichia coli* (Hufnagel et al., 2014; Raterman et al., 2013; Sanchez-Torres et al., 2011), *Klebsiella pneumonia* (Huertas et al., 2014) and *Yersinia pestis* (Ren et al., 2014). YfiN is an integral inner-membrane protein with two potential transmembrane helices, a periplasmic Per-Arnt-Sim (PAS) domain, and cytosolic domains containing a HAMP domain (mediate input-output signaling in histidine kinases, adenylyl cyclases, methyl-accepting chemotaxis proteins, and phosphatases) and a C-terminal GGDEF domain indicating a DGC’s function (Giardina et al., 2013; Malone et al., 2010). YfiN is repressed by specific interaction between its periplasmic PAS domain and the periplasmic protein YfiR (Malone et al., 2010). YfiB is an OmpA/Pal-like outer-membrane lipoprotein (Parsons et al., 2006) that can activate YfiN by sequestering YfiR (Malone et al., 2010) in an unknown manner. Whether YfiB directly recruits YfiR or recruits YfiR via a third partner is an open question. After the sequestration of YfiR by YfiB, the c-di-GMP produced by activated YfiN increases the biosynthesis of the Pel and Psl EPSs, resulting in the appearance of the SCV phenotype, which indicates enhanced biofilm formation (Malone et al., 2010).

It has been reported that the activation of YfiN may be induced by redox-driven misfolding of YfiR (Giardina et al., 2013; Malone et al., 2012; Malone et al., 2010). It is also proposed that the sequestration of YfiR by YfiB can be induced by certain YfiB-mediated cell wall stress, and mutagenesis studies revealed a number of activation residues of YfiB that were located in close proximity to the predicted first helix of the periplasmic domain (Malone et al., 2012). In addition, quorum sensing-related dephosphorylation of the PAS domain of YfiN may also be involved in the regulation (Ueda and Wood, 2009; Xu et al., 2015). Recently, we solved the crystal structure of YfiR in both the non-oxidized and the oxidized states, revealing breakage/formation of one disulfide bond (Cys71-Cys110) and local conformational change around the other one (Cys145-Cys152), indicating that Cys145-Cys152 plays an important role in maintaining the correct folding of YfiR (Yang et al., 2015).

In the present study, we solved the crystal structures of an N-terminal truncated form of YfiB (34–168) and YfiR in complex with an active mutant YfiB^L43P^. Most recently, Li and coworkers reported the crystal structures of YfiB (27–168) alone and YfiR^C71S^ in complex with YfiB (59–168) (Li et al., 2015). Compared with the reported complex structure, YfiB^L43P^ in our YfiB-YfiR complex structure has additional visible N-terminal residues 44–58 that are shown to play essential roles in YfiB activation and biofilm formation. Therefore, we are able to visualize the detailed allosteric arrangement of the N-terminal structure of YfiB and its important role in YfiB-YfiR interaction. In addition, we found that the YfiB^L43P^ shows a much higher PG-binding affinity than wild-type YfiB, most likely due to its more compact PG-binding pocket. Moreover, we found that Vitamin B6 (VB6) or L-Trp can bind YfiR with an affinity in the ten millimolar range. Together with functional data, these results provide new mechanistic insights into how activated YfiB sequesters YfiR and releases the suppression of YfiN. These findings may facilitate the development and optimization of anti-biofilm drugs for the treatment of chronic infections.

## RESULTS

### Overall structure of YfiB

We obtained two crystal forms of YfiB (residues 34–168, lacking the signal peptide from residues 1–26 and periplasmic residues 27–33), crystal forms I and II, belonging to space groups P2_1_ and P4_1_, respectively.

**Figure 1:**
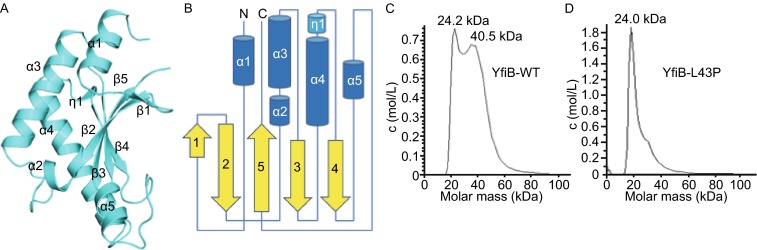
Overall structure of YfiB. (A) The overall structure of the YfiB monomer. (B) A topology diagram of the YfiB monomer. (C and D) The analytical ultracentrifugation experiment results for the wild-type YfiB and YfiB^L43P^

**Figure 2:**
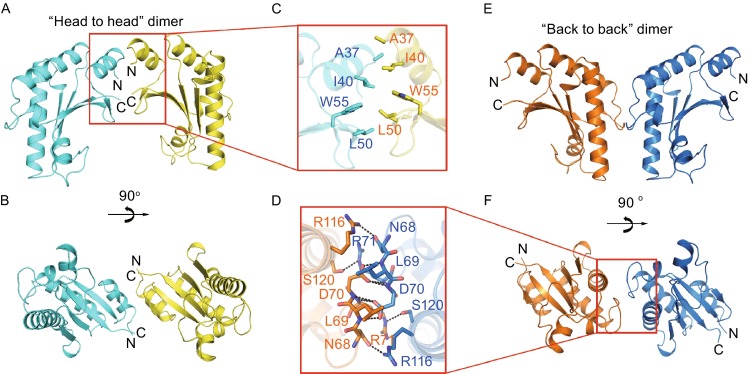
Two dimeric types of YfiB dimer. (A–C) The “head to head” dimer. (D–F) The “back to back” dimer. (A) and (E) indicate the front views of the two dimers, (B) and (F) indicate the top views of the two dimers, and (C) and (D) indicate the details of the two dimeric interfaces

The crystal structure of YfiB monomer consists of a five-stranded β-sheet (β1-2-5-3-4) flanked by five α-helices (α1–5) on one side. In addition, there is a short helix turn connecting the β4 strand and α4 helix (Fig. 1A and 1B). Each crystal form contains three different dimeric types of YfiB, two of which are present in both, suggesting that the rest of the dimeric types may result from crystal packing. Here, we refer to the two dimeric types as “head to head” and “back to back” according to the interacting mode (Fig. 2A and 2E), with the total buried surface areas being 316.8 Å^2^ and 554.3 Å^2^, respectively.

The “head to head” dimer exhibits a clamp shape. The dimerization occurs mainly via hydrophobic interactions formed by A37 and I40 on the α1 helices, L50 on the β1 strands, and W55 on the β2 strands of both molecules, making a hydrophobic interacting core (Fig. 2A–C).

The “back to back” dimer presents a Y shape. The dimeric interaction is mainly hydrophilic, occurring among the main-chain and side-chain atoms of N68, L69, D70 and R71 on the α2-α3 loops and R116 and S120 on the α4 helices of both molecules, resulting in a complex hydrogen bond network (Fig. 2D–F).

### The YfiB-YfiR interaction

**Figure 3:**
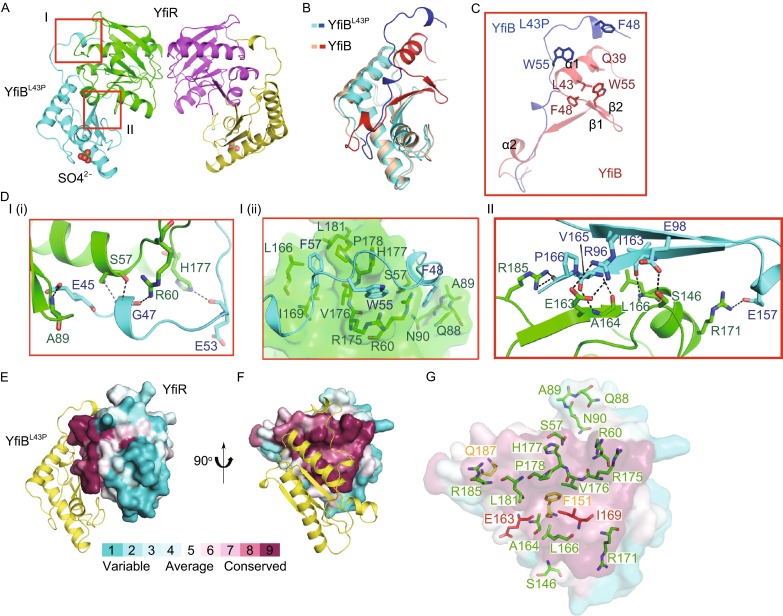
Overall structure of the YfiB-YfiR complex and the conserved surface in YfiR. (A) The overall structure of the YfiB-YfiR complex. The YfiB^L43P^ molecules are shown in cyan and yellow. The YfiR molecules are shown in green and magenta. Two interacting regions are highlighted by red rectangles. (B) Structural superposition of apo YfiB and YfiR-bound YfiB^L43P^. To illustrate the differences between apo YfiB and YfiR-bound YfiB^L43P^, the apo YfiB is shown in pink, except residues 34–70 are shown in red, whereas the YfiR-bound YfiB^L43P^ is shown in cyan, except residues 44–70 are shown in blue. (C) Close-up view of the differences between apo YfiB and YfiR-bound YfiB^L43P^. The residues proposed to contribute to YfiB activation are illustrated in sticks. The key residues in apo YfiB are shown in red and those in YfiB^L43P^ are shown in blue. (D) Close-up views showing interactions in regions I and II. YfiB^L43P^ and YfiR are shown in cyan and green, respectively. (E and F) The conserved surface in YfiR contributes to the interaction with YfiB. (G) The residues of YfiR responsible for interacting with YfiB are shown in green sticks, and the proposed YfiN-interacting residues are shown in yellow sticks. The red sticks, which represent the YfiB-interacting residues, are also responsible for the proposed interactions with YfiN

To gain structural insights into the YfiB-YfiR interaction, we co-expressed YfiB (residues 34–168) and YfiR (residues 35–190, lacking the signal peptide), but failed to obtain the complex, in accordance with a previous report in which no stable complex of YfiB-YfiR was observed (Malone et al., 2012). It has been reported that single mutants of Q39, L43, F48 and W55 contribute to YfiB activation leading to the induction of the SCV phenotype in *P. aeruginosa* PAO1 (Malone et al., 2012). It is likely that these residues may be involved in the conformational changes of YfiB that are related to YfiR sequestration (Fig. 3C). Therefore, we constructed two such single mutants of YfiB (YfiB^L43P^ and YfiB^F48S^). As expected, both mutants form a stable complex with YfiR. Finally, we crystalized YfiR in complex with the YfiB^L43P^ mutant and solved the structure at 1.78 Å resolution by molecular replacement using YfiR and YfiB as models.

The YfiB-YfiR complex is a 2:2 heterotetramer (Fig. 3A) in which the YfiR dimer is clamped by two separated YfiB^L43P^ molecules with a total buried surface area of 3161.2 Å^2^. The YfiR dimer in the complex is identical to the non-oxidized YfiR dimer alone (Yang et al., 2015), with only Cys145-Cys152 of the two disulfide bonds well formed, suggesting Cys71-Cys110 disulfide bond formation is not essential for forming YfiB-YfiR complex. The N-terminal structural conformation of YfiB^L43P^, from the foremost N-terminus to residue D70, is significantly altered compared with that of the apo YfiB. The majority of the α1 helix (residues 34–43) is invisible on the electron density map, and the α2 helix and β1 and β2 strands are rearranged to form a long loop containing two short α-helix turns (Fig. 3B and 3C), thus embracing the YfiR dimer. The observed changes in conformation of YfiB and the results of mutagenesis suggest a mechanism by which YfiB sequesters YfiR.

The YfiB-YfiR interface can be divided into two regions (Fig. 3A and 3D). Region I is formed by numerous main-chain and side-chain hydrophilic interactions between residues E45, G47 and E53 from the N-terminal extended loop of YfiB and residues S57, R60, A89 and H177 from YfiR (Fig. 3D-I(i)). Additionally, three hydrophobic anchoring sites exist in region I. The residues F48 and W55 of YfiB are inserted into the hydrophobic cores mainly formed by the main chain and side chain carbon atoms of residues S57/Q88/A89/N90 and R60/R175/H177 of YfiR, respectively; and F57 of YfiB is inserted into the hydrophobic pocket formed by L166/I169/V176/P178/L181 of YfiR (Fig. 3D-I(ii)). In region II, the side chains of R96, E98 and E157 from YfiB interact with the side chains of E163, S146 and R171 from YfiR, respectively. Additionally, the main chains of I163 and V165 from YfiB form hydrogen bonds with the main chains of L166 and A164 from YfiR, respectively, and the main chain of P166 from YfiB interacts with the side chain of R185 from YfiR (Fig. 3D-II). These two regions contribute a robust hydrogen-bonding network to the YfiB-YfiR interface, resulting in a tightly bound complex.

Based on the observations that two separated YfiB^L43P^ molecules form a 2:2 complex structure with YfiR dimer, we performed an analytical ultracentrifugation experiment to check the oligomeric states of wild-type YfiB and YfiB^L43P^. The results showed that wild-type YfiB exists in both monomeric and dimeric states in solution, while YfiB^L43P^ primarily adopts the monomer state in solution (Fig. 1C–D). This suggests that the N-terminus of YfiB plays an important role in forming the dimeric YfiB in solution and that the conformational change of residue L43 is associated with the stretch of the N-terminus and opening of the dimer. Therefore, it is possible that both dimeric types might exist in solution. For simplicity, we only discuss the “head to head” dimer in the following text.

### The PG-binding site of YfiB

**Figure 4:**
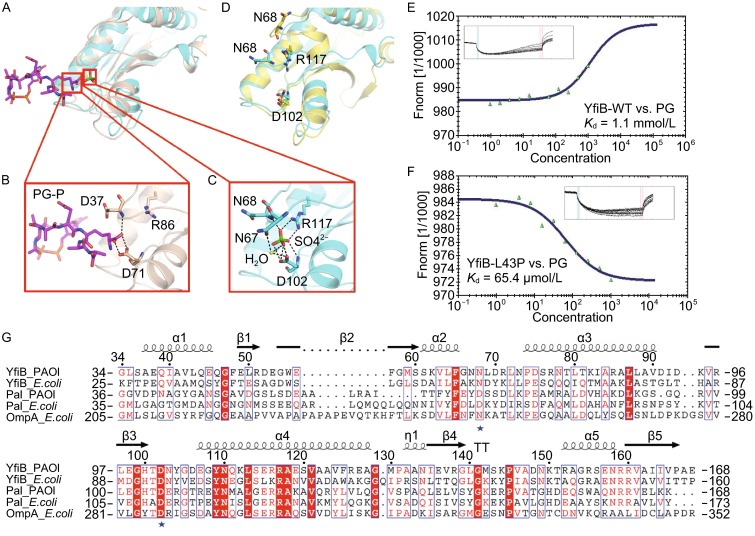
The PG-binding site in YfiB. (A) Structural superposition of the PG-binding sites of the *H. influenzae* Pal/PG-P complex and YfiR-bound YfiB^L43P^ complexed with sulfate ions. (B) Close-up view showing the key residues of Pal interacting with the *m*-Dap5 *ε*-carboxylate group of PG-P. Pal is shown in wheat and PG-P is in magenta. (C) Close-up view showing the key residues of YfiR-bound YfiB^L43P^ interacting with a sulfate ion. YfiR-bound YfiB^L43P^ is shown in cyan; the sulfate ion, in green; and the water molecule, in yellow. (D) Structural superposition of the PG-binding sites of apo YfiB and YfiR-bound YfiB^L43P^, the key residues are shown in stick. Apo YfiB is shown in yellow and YfiR-bound YfiB^L43P^ in cyan. (E and F) MST data and analysis for binding affinities of (E) YfiB wild-type and (F) YfiB^L43P^ with PG. (G) The sequence alignment of *P. aeruginosa* and *E. coli* sources of YfiB, Pal and the periplasmic domain of OmpA

PG-associated lipoprotein (Pal) is highly conserved in Gram-negative bacteria and anchors to the outer membrane through an N-terminal lipid attachment and to PG layer through its periplasmic domain, which is implicated in maintaining outer membrane integrity. Previous homology modeling studies suggested that YfiB contains a Pal-like PG-binding site (Parsons et al., 2006), and the mutation of two residues at this site, D102 and G105, reduces the ability for biofilm formation and surface attachment (Malone et al., 2012). In the YfiB-YfiR complex, one sulfate ion is found at the bottom of each YfiB^L43P^ molecule (Fig. 3A) and forms a strong hydrogen bond with D102 of YfiB^L43P^ (Fig. 4A and 4C). Structural superposition between YfiB^L43P^ and Haemophilus influenzae Pal complexed with biosynthetic peptidoglycan precursor (PG-P), UDP-N-acetylmuramyl-L-Ala-α-D-Glu-m-Dap-D-Ala-D-Ala (m-Dap is meso-diaminopimelate) (PDB code: 2aiz) (Parsons et al., 2006), revealed that the sulfate ion is located at the position of the m-Dap5 ϵ-carboxylate group in the Pal/PG-P complex (Fig. 4A). In the Pal/PG-P complex structure, the m-Dap5 ϵ-carboxylate group interacts with the side-chain atoms of D71 and the main-chain amide of D37 (Fig. 4B). Similarly, in the YfiR-bound YfiB^L43P^ structure, the sulfate ion interacts with the side-chain atoms of D102 (corresponding to D71 in Pal) and R117 (corresponding to R86 in Pal) and the main-chain amide of N68 (corresponding to D37 in Pal). Moreover, a water molecule was found to bridge the sulfate ion and the side chains of N67 and D102, strengthening the hydrogen bond network (Fig. 4C). In addition, sequence alignment of YfiB with Pal and the periplasmic domain of OmpA (proteins containing PG-binding site) showed that N68 and D102 are highly conserved (Fig. 4G, blue stars), suggesting that these residues contribute to the PG-binding ability of YfiB.

Interestingly, superposition of apo YfiB with YfiR-bound YfiB^L43P^ revealed that the PG-binding region is largely altered mainly due to different conformation of the N68 containing loop. Compared to YfiB^L43P^, the N68-containing loop of the apo YfiB flips away about 7 Å, and D102 and R117 swing slightly outward; thus, the PG-binding pocket is enlarged with no sulfate ion or water bound (Fig. 4D). Therefore, we proposed that the PG-binding ability of inactive YfiB might be weaker than that of active YfiB. To validate this, we performed a microscale thermophoresis (MST) assay to measure the binding affinities of PG to wild-type YfiB and YfiB^L43P^, respectively. The results indicated that the PG-binding affinity of YfiB^L43P^ is 65.5 μmol/L, which is about 16-fold stronger than that of wild-type YfiB (*K*_d_ = 1.1 mmol/L) (Fig. 4E–F). As the experiment is performed in the absence of YfiR, it suggests that an increase in the PG-binding affinity of YfiB is not a result of YfiB-YfiR interaction and is highly coupled to the activation of YfiB characterized by a stretched N-terminal conformation.

### The conserved surface in YfiR is functional for binding YfiB and YfiN

Calculation using the ConSurf Server (http://consurf.tau.ac.il/), which estimates the evolutionary conservation of amino acid positions and visualizes information on the structure surface, revealed a conserved surface on YfiR that contributes to the interaction with YfiB (Fig. 3E and 3F). Interestingly, the majority of this conserved surface contributes to the interaction with YfiB (Fig. 3E and 3F). Malone JG et al. have reported that F151, E163, I169 and Q187, located near the C-terminus of YfiR, comprise a putative YfiN binding site (Malone et al., 2012). Interestingly, these residues are part of the conserved surface of YfiR (Fig. 3G). F151, E163 and I169 form a hydrophobic core while, Q187 is located at the end of the α6 helix. E163 and I169 are YfiB-interacting residues of YfiR, in which E163 forms a hydrogen bond with R96 of YfiB (Fig. 3D-II) and I169 is involved in forming the L166/I169/V176/P178/L181 hydrophobic core for anchoring F57 of YfiB (Fig. 3D-I(ii)). Collectively, a part of the YfiB-YfiR interface overlaps with the proposed YfiR-YfiN interface, suggesting alteration in the association-disassociation equilibrium of YfiR-YfiN and hence the ability of YfiB to sequester YfiR.

### YfiR binds small molecules

Previous studies indicated that YfiR constitutes a YfiB-independent sensing device that can activate YfiN in response to the redox status of the periplasm, and we have reported YfiR structures in both the non-oxidized and the oxidized states earlier, revealing that the Cys145-Cys152 disulfide bond plays an essential role in maintaining the correct folding of YfiR (Yang et al., 2015). However, whether YfiR is involved in other regulatory mechanisms is still an open question.

**Figure 5:**
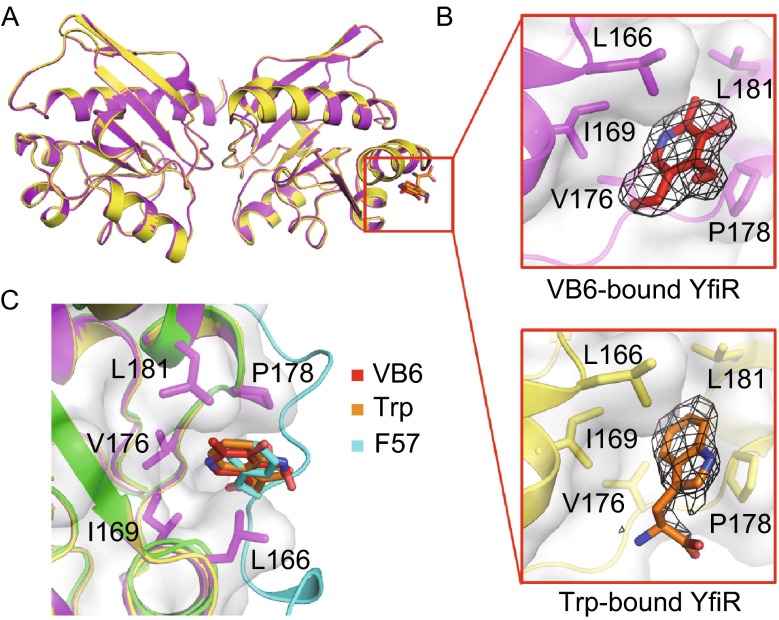
Overall Structures of VB6-bound and Trp-bound YfiR. (A) Superposition of the overall structures of VB6-bound and Trp-bound YfiR. (B) Close-up views showing the key residues of YfiR that bind VB6 and L-Trp. The electron densities of VB6 and Trp are countered at 3.0σ and 2.3σ, respectively, in |*F*o|-|*F*c| maps. (C) Superposition of the hydrophobic pocket of YfiR with VB6, L-Trp and F57 of YfiB

Intriguingly, a Dali search (Holm and Rosenstrom, 2010) indicated that the closest homologs of YfiR shared the characteristic of being able to bind several structurally similar small molecules, such as L-Trp, L-Phe, B-group vitamins and their analogs, encouraging us to test whether YfiR can recognize these molecules. For this purpose, we co-crystallized YfiR or soaked YfiR crystals with different small molecules, including L-Trp and B-group vitamins. Fortunately, we found obvious small-molecule density in the VB6-bound and Trp-bound YfiR crystal structures (Fig. 5A and 5B), and in both structures, the YfiR dimers resemble the oxidized YfiR structure in which both two disulfide bonds are well formed (Yang et al., 2015).

**Figure 6:**
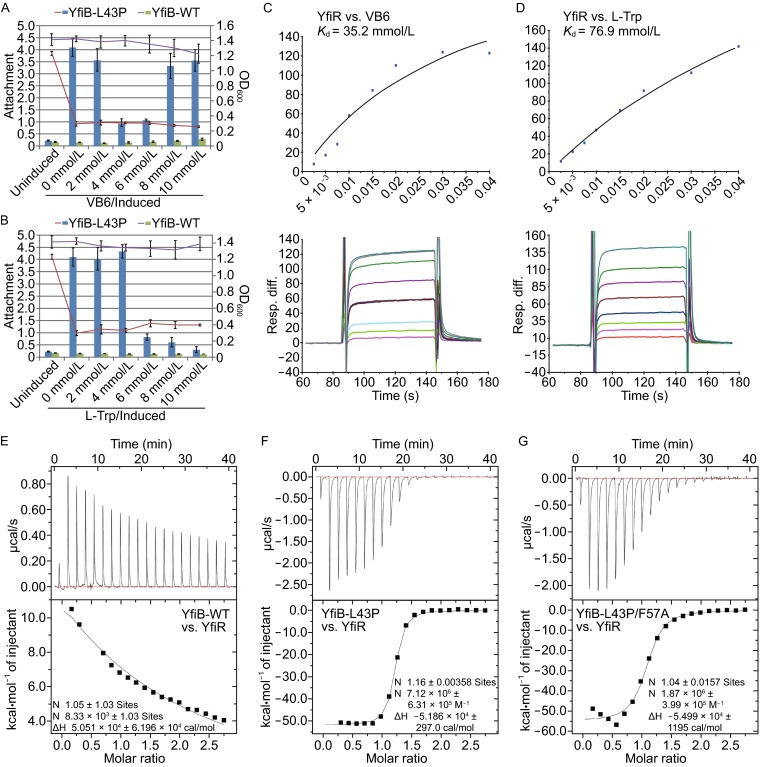
Functional analysis of VB6 and L-Trp. (A and B) The effect of increasing concentrations of VB6 or L-Trp on YfiB^L43P^-induced attachment (bars). The relative optical density is represented as curves. Wild-type YfiB is used as negative control. (C and D) BIAcore data and analysis for binding affinities of (C) VB6 and (D) L-Trp with YfiR. (E–G) ITC data and analysis for titration of (E) YfiB wild-type, (F) YfiB^L43P^, and (G) YfiB^L43P/F57A^ into YfiR

Structural analyses revealed that the VB6 and L-Trp molecules are bound at the periphery of the YfiR dimer, but not at the dimer interface. Interestingly, VB6 and L-Trp were found to occupy the same hydrophobic pocket, formed by L166/I169/V176/P178/L181 of YfiR, which is also a binding pocket for F57 of YfiB, as observed in the YfiB-YfiR complex (Fig. 5C). To evaluate the importance of F57 in YfiB^L43P^-YfiR interaction, the binding affinities of YfiB^L43P^ and YfiB^L43P/F57A^ for YfiR were measured by isothermal titration calorimetry (ITC). The results showed K_d_ values of 1.4 × 10^−7^ mol/L and 5.3 × 10^−7^ mol/L for YfiB^L43P^ and YfiB^L43P/F57A^, respectively, revealing that the YfiB^L43P/F57A^ mutant caused a 3.8-fold reduction in the binding affinity compared with the YfiB^L43P^ mutant (Fig. 6F and 6G).

In parallel, to better understand the putative functional role of VB6 and L-Trp, *yfiB* was deleted in a PAO1 wild-type strain, and a construct expressing the YfiB^L43P^ mutant was transformed into the PAO1 Δ*yfiB* strain to trigger YfiB^L43P^-induced biofilm formation. Growth and surface attachment assays were carried out for the *yfiB*-L43P strain in the presence of increasing concentrations of VB6 or L-Trp. As shown in Fig. 6A and 6B, the over-expression of YfiB^L43P^ induced strong surface attachment and much slower growth of the *yfiB*-L43P strain, and as expected, a certain amount of VB6 or L-Trp (4–6 mmol/L for VB6 and 6–10 mmol/L for L-Trp) could reduce the surface attachment. Interestingly, at a concentration higher than 8 mmol/L, VB6 lost its ability to inhibit biofilm formation, implying that the VB6-involving regulatory mechanism is highly complicated and remains to be further investigated.

Of note, both VB6 and L-Trp have been reported to correlate with biofilm formation in certain Gram-negative bacteria (Grubman et al., 2010; Shimazaki et al., 2012). In *Helicobacter pylori* in particular, VB6 biosynthetic enzymes act as novel virulence factors, and VB6 is required for full motility and virulence (Grubman et al., 2010). In *E.* *coli*, mutants with decreased tryptophan synthesis show greater biofilm formation, and matured biofilm is degraded by L-tryptophan addition (Shimazaki et al., 2012). However, the detailed mechanism remains elusive.

To answer the question whether competition of VB6 or L-Trp for the YfiB F57-binding pocket of YfiR plays an essential role in inhibiting biofilm formation, we measured the binding affinities of VB6 and L-Trp for YfiR via BIAcore experiments. The results showed relatively weak *K*_d_ values of 35.2 mmol/L and 76.9 mmol/L for VB6 and L-Trp, respectively (Fig. 6C and 6D). Based on our results, we concluded that VB6 or L-Trp can bind to YfiR, however, VB6 or L-Trp alone may have little effects in interrupting the YfiB-YfiR interaction, the mechanism by which VB6 or L-Trp inhibits biofilm formation remains unclear and requires further investigation.

## DISCUSSION

Previous studies suggested that in response to cell stress, YfiB in the outer membrane sequesters the periplasmic protein YfiR, releasing its inhibition of YfiN on the inner membrane and thus inducing the diguanylate cyclase activity of YfiN to allow c-di-GMP production (Giardina et al., 2013; Malone et al., 2012; Malone et al., 2010). However, the pattern of interaction between these proteins and the detailed regulatory mechanism remain unknown due to a lack of structural information.

Here, we report the crystal structures of YfiB alone and an active mutant YfiB^L43P^ in complex with YfiR, indicating that YfiR forms a 2:2 complex with YfiB via a region composed of conserved residues. Our structural data analysis shows that the activated YfiB has an N-terminal portion that is largely altered, adopting a stretched conformation compared with the compact conformation of the apo YfiB. The apo YfiB structure constructed beginning at residue 34 has a compact conformation of approximately 45 Å in length. In addition to the preceding 8 aa loop (from the lipid acceptor Cys26 to Gly34), the full length of the periplasmic portion of apo YfiB can reach approximately 60 Å. It was reported that the distance between the outer membrane and the cell wall is approximately 50 Å and that the thickness of the PG layer is approximately 70 Å (Matias et al., 2003). Thus, YfiB alone represents an inactive form that may only partially insert into the PG matrix. By contrast, YfiR-bound YfiB^L43P^ (residues 44–168) has a stretched conformation of approximately 55 Å in length. In addition to the 17 preceding intracellular residues (from the lipid acceptor Cys26 to Leu43), the length of the intracellular portion of active YfiB may extend over 100 Å, assuming a fully stretched conformation. Provided that the diameter of the widest part of the YfiB dimer is approximately 64 Å, which is slightly smaller than the smallest diameter of the PG pore (70 Å) (Meroueh et al., 2006), the YfiB dimer should be able to penetrate the PG layer.

**Figure 7:**
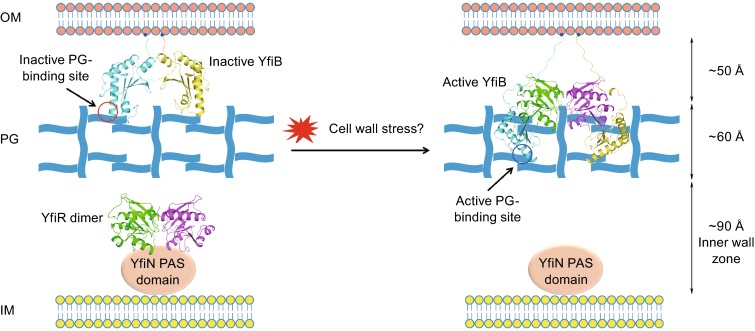
Regulatory model of the YfiBNR tripartite system. The periplasmic domain of YfiB and the YfiB-YfiR complex are depicted according to the crystal structures. The lipid acceptor Cys26 is indicated as blue ball. The loop connecting Cys26 and Gly34 of YfiB is modeled. The PAS domain of YfiN is shown as pink oval. Once activated by certain cell stress, the dimeric YfiB transforms from a compact conformation to a stretched conformation, allowing the periplasmic domain of the membrane-anchored YfiB to penetrate the cell wall and sequester the YfiR dimer, thus relieving the repression of YfiN

These results, together with our observation that activated YfiB has a much higher cell wall binding affinity, and previous mutagenesis data showing that (1) both PG binding and membrane anchoring are required for YfiB activity and (2) activating mutations possessing an altered N-terminal loop length are dominant over the loss of PG binding (Malone et al., 2012), suggest an updated regulatory model of the YfiBNR system (Fig. 7). In this model, in response to a particular cell stress that is yet to be identified, the dimeric YfiB is activated from a compact, inactive conformation to a stretched conformation, which possesses increased PG binding affinity. This allows the C-terminal portion of the membrane-anchored YfiB to reach, bind and penetrate the cell wall and sequester the YfiR dimer. The YfiBNR system provides a good example of a delicate homeostatic system that integrates multiple signals to regulate the c-di-GMP level. Homologs of the YfiBNR system are functionally conserved in *P. aeruginosa* (Malone et al., 2012; Malone et al., 2010), *E. coli* (Hufnagel et al., 2014; Raterman et al., 2013; Sanchez-Torres et al., 2011), *K. pneumonia* (Huertas et al., 2014) and *Y. pestis* (Ren et al., 2014), where they affect c-di-GMP production and biofilm formation. The mechanism by which activated YfiB relieves the repression of YfiN may be applicable to the YfiBNR system in other bacteria and to analogous outside-in signaling for c-di-GMP production, which in turn may be relevant to the development of drugs that can circumvent complicated antibiotic resistance.

## MATERIALS AND METHODS

### Protein expression and purification

*P. aeruginosa* YfiR (residues 35–190, lacking the predicted N-terminal periplasmic localization signaling peptide) and YfiB (residues 34–168, lacking the signal peptide from residues 1–26 and periplasmic residues 27–33) were cloned into ORF1 of the pETDuet-1 (Merck Millipore, Darmstadt, Germany) vector via the *Bam*HI and *Hin*dIII restriction sites, with a constructed N-terminal His_6_ and a TEV cleavage site, respectively. In addition, YfiB (residues 34–168) was ligated into the *Nde*I and *Xho*I restriction sites of ORF2 in the previously constructed YfiR expression vector. Site-directed mutagenesis was carried out using a QuikChange kit (Agilent Technologies, Santa Clara, CA), following the manufacturer’s instructions.

The proteins were over-expressed in the *E. coli* BL21-CodonPlus(DE3)-RIPL strain. Protein expression was induced by adding 0.5–1 mmol/L IPTG at an OD600 of approximately 0.8. The cell cultures were then incubated for an additional 4.5 h at 37°C. The cells were subsequently harvested by centrifugation and stored at −80°C.

Cell suspensions were thawed and homogenized using a high-pressure homogenizer (JNBIO, Beijing, China). YfiR was first purified by Ni affinity chromatography and then incubated with His_6_-tagged TEV protease overnight. The His_6_-TEV cleavage site was subsequently removed by incubation with Ni-NTA resin. Finally, YfiR was purified with a HiTrap S^TM^ column (GE Healthcare), followed by a Superdex 200 (GE Healthcare) column. YfiB was purified with Ni affinity chromatography, followed by a Superdex 200 (GE Healthcare) column. The YfiB-YfiR complex was first purified by Ni affinity chromatography, then by a Superdex 200 (GE Healthcare) column, and finally by a HiTrap S^TM^ column (GE Healthcare). All of the purified fractions were collected and concentrated to ~40 mg/mL in 20 mmol/L Tris-HCl (pH 8.0) and 200 mmol/L NaCl, frozen in liquid nitrogen and stored at −80°C.

### Crystallization and data collection

Crystal screening was performed with commercial screening kits (Hampton Research, CA, USA) using the sitting-drop vapor diffusion method, and positive hits were optimized using the hanging-drop vapor diffusion method at 293 K. Crystals of the YfiB protein were obtained and optimized in buffer containing 0.2 mol/L lithium sulfate monohydrate, 0.1 mol/L Tris-HCl (pH 8.0) and 30% *w*/*v* polyethylene glycol 4000. After being soaked for a few seconds in cryoprotection solution (well solution complemented with 25% xylitol), the crystals were cooled by plunging them into liquid nitrogen. Diffraction-quality crystals of the YfiB-YfiR complex were grown in buffer containing 0.2 mol/L ammonium sulfate, 0.1 mol/L Tris-HCl (pH 8.0) and 12% *w*/*v* polyethylene glycol 8000. The crystals were cryoprotected with 8% (*w*/*v*) polyethylene glycol 8000 and 0.1 mol/L Tris-HCl (pH 7.5) supplemented with saturated sucrose prior to being flash frozen. Crystals of the native YfiR were obtained and optimized in 0.1 mol/L HEPES (pH 7.5) and 1.8 mol/L ammonium sulfate. VB6-bound YfiR crystals were obtained by soaking the native YfiR crystals in 2 mmol/L VB6 molecules. Trp-bound YfiR crystals were obtained by co-crystalizing the YfiR protein and 4 mmol/L L-Trp molecules in 0.2 mol/L NaCl, 0.1 mol/L BIS-TRIS (pH 5.5), and 25% *w*/*v* polyethylene glycol 3350. For cryoprotection, both the VB6-bound and the L-Trp-bound YfiR crystals were soaked in 2.5 mol/L lithium sulfate monohydrate for a few seconds before data collection. Diffraction data for the YfiB crystal belonging to space group P2_1_ was collected in house, the data for the YfiB crystal belonging to space group P4_1_ and for the Trp-bound YfiR crystal were collected on beamline BL17U at the Shanghai Synchrotron Radiation Facility (SSRF), and the data for the VB6-bound YfiR crystal were collected on beamline BL18U at SSRF. Finally, the data for the YfiB-YfiR complex crystal were collected on beamline BL-1A at the Photon Factory in Japan. The diffraction data were processed with the HKL2000 software program (Otwinowski and Minor, 1997).

### Structure determination and refinement

The two YfiB crystal structures respectively belonging to space groups P2_1_ and P4_1_ were both solved by molecular replacement (Lebedev et al., 2008) using the putative MotB-like protein DVU_2228 from *D. vulgaris* as a model (PDB code: 3khn) at 2.15 Å and 2.8 Å resolution, respectively. Both the VB6-bound and the Trp-bound YfiR crystals belonging to space group P4_3_2_1_2, with a dimer in the asymmetric unit, were solved by molecular replacement (Lebedev et al., 2008) using native YfiR as a model (PDB code: 4YN7) at 2.4 Å and 2.5 Å resolution, respectively. The YfiB-YfiR crystal belonging to space group P1, with a 2:2 heterotetramer in the asymmetric unit, was solved by molecular replacement using YfiR and YfiB as models. Electron density maps were calculated using PHENIX (Adams et al., 2010). Model building was performed using COOT (Emsley et al., 2010) and refined with PHENIX (Adams et al., 2010; Afonine et al., 2012). The final structures were analyzed with PROCHECK (Laskowski et al., 1993). Data collection and refinement statistics are presented in Table 1. The figures depicting structures were prepared using PyMOL (http://www.pymol.org). Atomic coordinates and structure factors have been deposited in the RCSB Protein Data Bank (http://www.pdb.org) under accession codes 5EAZ, 5EB0, 5EB1, 5EB2 and 5EB3.

**Table 1:** Data collection, phasing and refinement statistics

**Data collection**	YfiB (crystal form I)	YfiB (crystal form II)	VB6-bound YfiR	Trp-bound YfiR	YfiBL43P-YfiR
Space group	*P*21	*P*41	*P*43212	*P*43212	*P*1
Wavelength (Å)	1.54187	0.9791	0.97861	0.9791	1.10000
Resolution (Å)^a^	50.0–2.15 (2.19–2.15)	50.0–2.80 (2.85–2.8)	50.0–2.4 (2.44–2.4)	50.0–2.5 (2.54–2.5)	50–1.78 (1.86–1.78)
Cell dimensions					
a, b, c (Å)	65.85, 90.45, 66.30	46.95, 46.95, 154.24	120.24, 120.24, 84.99	120.88, 120.88, 88.46	49.50, 58.57, 69.86
α, β, γ (°)	90, 113.87, 90	90, 90, 90	90, 90, 90	90, 90, 90	72.93, 96.98, 90.19
Unique reflections	37,625 (1866)	8,105 (412)	24,776 (1202)	23170 (1132)	67,774 (6615)
* I/*σ*I*	19.59 (2.62)	12.36 (4.15)	20.17 (2.4)	39.5 (4.68)	17.75 (1.89)
Completeness (%)	97.1 (95.4)	97.8 (100)	99.1 (98.8)	99.9 (100)	96.5 (94.6)
*R*_merge_ (%)	6.5 (44.5)	14.6 (49.7)	8.9 (56.8)	9.4 (89.2)	5.6 (46.3)
* R*_meas_ (%)	7.4 (51.6)	15.4 (52.0)	9.6 (61.7)	9.6 (90.8)	6.6 (55.1)
CC1/2^b^	0.747	0.952	0.899	0.974	0.849
**Refinement**					
* R*_work_ (%)	20.14	19.17	17.82	18.66	17.90
* R*_free_(%)	26.29	26.49	19.81	23.05	20.61
Average B factors (Å^2^)					
Protein	25.54	42.70	38.68	35.03	32.54
VB6	-	-	44.08	-	-
Trp	-	-	-	87.51	-
SO_4_^2−^	37.16	66.52	51.55	41.93	45.51
H_2_O	32.91	36.09	40.58	34.75	43.52
Root mean square deviations					
Bond lengths (Å)	0.009	0.009	0.007	0.007	0.007
Bond angles (°)	1.085	1.132	1.021	0.977	1.110
Ramachandran plot					
Most favored (%)	92.6	87.7	96.5	98.1	94.2
Additionally allowed (%)	7.4	12.3	3.5	1.9	5.8
Generously allowed (%)	0	0	0	0	0
Disallowed	0	0	0	0	0

^a^ Numbers in parentheses are for the highest resolution shell

^b^ The values of CC1/2 are for the highest resolution shell

### Analytical ultracentrifugation

Sedimentation velocity measurements were performed on a Beckman ProteomeLab XL-I at 25°C. All protein samples were diluted to an OD_280_ of 0.7 in 20 mmol/L Tris (pH 8.0) and 200 mmol/L NaCl. Data were collected at 60,000 rpm. (262,000 ×*g*) every 3 min at a wavelength of 280 nm. Interference sedimentation coefficient distributions, or c(M), were calculated from the sedimentation velocity data using SEDFIT (Schuck, 2000).

### PG preparation

PG was extracted from the *E. coli* DH5α strain by following a method described previously (Desmarais et al., 2014). Briefly, cells were cultured until they reached an OD_600_ of 0.7–0.8 and then collected at 5,000 ×*g*, 4°C. The collected bacteria were dripped into the boiling 6% (*w*/*v*) SDS and stirred at 500 rpm in a boiling water bath for 3 h before incubating overnight at room temperature. The large PG polymers were collected by ultracentrifugation at 130,000 ×*g* for 1 h at room temperature and washed repeatedly to remove SDS. The pellet was treated with Pronase E (200 μg/mL final concentration) for 3 h at 60°C followed by SDS to remove contaminating proteins and washed three times to remove the SDS by ultracentrifugation. Next, the samples were treated with lysozyme (200 μg/mL final concentration) for 16 h at 37°C. Finally, the purified PG is obtained by treating the samples in a boiling water bath for 10 min and centrifuging it at 13,000 ×*g* to remove the contaminating lysozyme.

### Microscale thermophoresis (MST)

Purified YfiB wild-type and it mutant YfiB^L43P^ were fluorescently labeled using the NanoTemper blue protein-labeling kit according to the manufacturer’s protocol. This resulted in coupling of the fluorescent dye NT-495. PG was titrated in 1:1 dilutions starting at 1 mmol/L. To determine of the *K*_d_ values, 10 μL labeled protein was mixed with 10 μL PG at various concentrations in Hepes buffer (20 mmol/L Hepes, 200 mmol/L NaCl, 0.005% Tween-20, pH 7.5). After 10 min of incubation, all binding reaction mixtures were loaded into the MST-grade glass capillaries (NanoTemper Technologies), and thermophoresis was measured with a NanoTemper Monolith-NT115 system (20% light-emitting diode, 20% IR laser power).

### Deletion of the *yfiB* genes

The *yfiB* deletion construct was produced by SOE-PCR (Hmelo et al., 2015) and contained homologous flanking regions to the target gene. This construct was ligated into the pEX18Gm vector between the *Hin*dIII and the KpnI sites. The resulting vector was then used to delete *yfiB* by two-step allelic exchange (Hmelo et al., 2015). After being introduced into PAO1 via biparental mating with *E. coli* SM10 (λpir), single crossovers were selected on Vogel-Bonner Minimal Medium (VBMM), which was used for counter-selection against *E. coli* (*P. aeruginosa* can utilize citrate as a sole carbon source and energy source, whereas *E. coli* cannot), containing 50 μg/mL gentamycin. Restreaking was then performed on no-salt Luria-Bertani (NSLB) agar that contained 15% sucrose to force the resolution of double crossovers. Deletion of *yfiB* in the strains was confirmed by colony PCR.

For complementation experiments, *yfiB* wild-type and L43P mutant genes were cloned into the pJN105 vector via the *Eco*RI and *Xba*I restriction sites, respectively. The plasmids were then individually transformed into the PAO1 Δ*yfiB* strain using the rapid electroporation method described in Choi KH et al. (Choi et al., 2006). Transformants were selected on LB plates containing 50 μg/mL gentamycin. For induction, arabinose was added to a final concentration of 0.2%.

### Attachment assays

The attachment assays were carried out using the MBEC^TM^ (Minimum Biofilm Eradication Concentration, Innovotech, Inc.) biofilm inoculator, which consists of a plastic lid with 96 pegs and 96 individual wells. The MBEC plates containing 150 μL LB medium/well were inoculated with 1% overnight cultures of the *yfiB*-L43P strain and incubated overnight at 37°C without shaking. VB6, L-Trp and arabinose were added as appropriate. The peg lids were washed with distilled water, and the attached cell material was then stained with 0.1% crystal violet solution (5% methanol, 5% isopropanol) before further washing to remove excess dye. The crystal violet was re-dissolved in 20% acetic acid solution, and the absorbance was measured at 600 nm. Assays were performed with 12 wells/strain and repeated independently for each experiment.

### BIAcore analysis

The interaction kinetics of YfiR with VB6 and L-Trp were examined on a SPR machine Biacore 3000 (GE Healthcare) at 25°C. The running buffer (20 mmol/L HEPES, pH 7.5, 150 mmol/L NaCl, 0.005% (*v*/*v*) Tween-20) was vacuum filtered, and degassed immediately prior to use. YfiR at 10 μg/mL in 10 mmol/L sodium acetate (pH 5.5) was immobilized to 3000 response units on the carboxymethylated dextran surface-modified chip (CM5 chip). The binding affinities were evaluated over a range of 2.5–40 mmol/L concentrations. Meanwhile, for both binding assays, the concentration of 10 mmol/L was repeated as an internal control. All of the data collected were analyzed using BIAevaluation software version 4.1.

### ITC assays

ITC experiments were performed in a buffer composed of 20 mmol/L Tris (pH 8.0) and 150 mmol/L NaCl at 25°C using an iTC200 calorimeter (GE Healthcare). YfiB wild-type or its mutants (YfiB^L43P^, YfiB^L43P/F57A^) (0.4 mmol/L, in the syringe) was titrated into YfiR (0.04 mmol/L, in the cell), respectively. The titration sequence included a single 0.5 µL injection, followed by 19 injections of 2 µL each, with a 2-min interval between injections and a stirring rate of 1000 rpm. The calorimetric data were then analyzed with OriginLab software (GE Healthcare).
